# Multiscale Homogenization Techniques for TPMS Foam Material for Biomedical Structural Applications

**DOI:** 10.3390/bioengineering10050515

**Published:** 2023-04-25

**Authors:** Ana Pais, Jorge Lino Alves, Renato Natal Jorge, Jorge Belinha

**Affiliations:** 1 INEGI—Institute of Science and Innovation in Mechanical and Industrial Engineering, Rua Dr. Roberto Frias, s/n, 4200-465 Porto, Portugal; 2 FEUP—Faculty of Engineering, University of Porto, Rua Dr. Roberto Frias, s/n, 4200-465 Porto, Portugal; 3 ISEP—School of Engineering, Polytechnic of Porto, Rua Dr. António Bernardino de Almeida, 431, 4249-015 Porto, Portugal

**Keywords:** homogenization, multiscale, biomedical application, triply periodic minimal surfaces, mechanical properties, femoral stem

## Abstract

Multiscale techniques, namely homogenization, result in significant computational time savings in the analysis of complex structures such as lattice structures, as in many cases it is inefficient to model a periodic structure in full detail in its entire domain. The elastic and plastic properties of two TPMS-based cellular structures, the gyroid, and the primitive surface are studied in this work through numerical homogenization. The study enabled the development of material laws for the homogenized Young’s modulus and homogenized yield stress, which correlated well with experimental data from the literature. It is possible to use the developed material laws to run optimization analyses and develop optimized functionally graded structures for structural applications or reduced stress shielding in bio-applications. Thus, this work presents a study case of a functionally graded optimized femoral stem where it was shown that the porous femoral stem built with Ti-6Al-4V can minimize stress shielding while maintaining the necessary load-bearing capacity. It was shown that the stiffness of cementless femoral stem implant with a graded gyroid foam presents stiffness that is comparable to that of trabecular bone. Moreover, the maximum stress in the implant is lower than the maximum stress in trabecular bone.

## 1. Introduction

Foam-like materials based on triply periodic minimal surfaces (TPMS) have a high potential in biomedical applications. Its highly porous configuration is beneficial for cell growth and proliferation, and thus, it becomes a valid alternative to design scaffolds for biomedical applications, such as bone tissue growth. The modification of the cell density allows adjusting the mechanical properties of the implant so as to avoid unwanted phenomena such as stress shielding, which occurs when the stiffness of the implant is much higher than the stiffness of the tissue it is replacing, as a consequence of the Wolff’s Law [1]. In comparison to other scaffold shapes, gyroid-based scaffolds present higher permeability than other triply periodic minimal surfaces (TPMS) [2]. Additionally, TPMS-based foams built using additive manufacturing technology have been shown to be able to achieve the necessary mechanical properties to replace human bone [3,4].

TPMS foams are the adaptation of the surface equation into a solid. The surface equation for different TPMS is the following, namely the gyroid (G) and the primitive (P):

(1) $$G:sin\left(\frac{2\pi x}{L}\right)cos\left(\frac{2\pi y}{L}\right)+sin\left(\frac{2\pi y}{L}\right)cos\left(\frac{2\pi z}{L}\right)+sin\left(\frac{2\pi z}{L}\right)cos\left(\frac{2\pi x}{L}\right)=t$$

(2) $$P:cos\left(\frac{2\pi x}{L}\right)+cos\left(\frac{2\pi y}{L}\right)+cos\left(\frac{2\pi z}{L}\right)=t$$

where *L* is the length of the unit cell. If *t* is equal to zero then the gyroid surface divides the space into two equal parts.

In this work, the term TPMS foam refers to the sheet foam variation of the TPMS. The surface may be adapted into a solid in two different manners, sheet foam or truss-like foam. The difference between sheet foams and truss-like foams consists in how the surface is converted to a solid. The surface divides the space into two parts, and if the space enclosed by one of the sides of the surface is chosen, a solid network is obtained. On the other hand, if the surface is “thickened”, a sheet network is obtained.

According to Al-Ketan et al. [5] sheet solid foams exhibit higher mechanical properties for the same apparent density as truss-like solids. In terms of surface area, which is also very relevant to the study of scaffold viability, sheet-like foams present higher surface area [6], because the surface is projected twice while the truss-like foam consists only of one repetition of the surface. It is also due to this that the gyroid sheet solid is also named double-gyroid [7].Addionally, TPMS based foam materials have been known to improve thermal properties such as conduction and convection [8,9].

**Figure 1:**
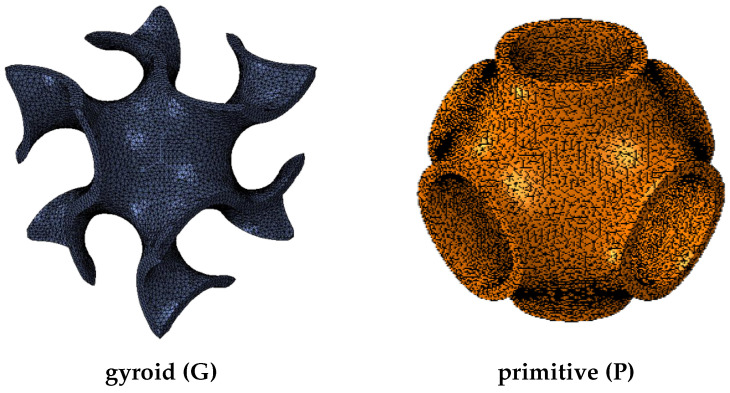
Sheet foams based on the gyroid and primitive surface.

An example of the two foams (gyroid and primitive) is shown in Figure 1.

### Lattice Materials for Tissue Engineering

Treatments for large bone defects in the future show significant potential when using scaffold-based bone tissue engineering.

Bone implants are one of the most commonly used devices in healthcare and also one of the riskiest, as revision surgeries are often necessary. The most common cause for revision surgery is the loosening of the implant due to bone resorption, which occurs as a consequence of the stress shielding phenomenon [10].

The 3D matrix known as a bone scaffold is what enables and promotes osteoinducible cells to connect to and proliferate on its surfaces [11]. Some of the main concerns in designing scaffold are its [11]:

1. biocompatibility;
2. biodegradability;
3. mechanical properties to bear weight during the amelioration period;
4. proper architecture in terms of porosity and pore sizes;
5. sterilibility without loss of bioactivity;
6. controlled deliverability of bioactive molecules or drugs;

Regarding mechanical properties, one can summarize the design of the bone scaffold into two main aspects: the stiffness of the scaffold, which must be low enough to allow for adequate load transfer to the bone; and strength, to withstand the naturally occurring loads in the human body. While bone presents anisotropy, there are no extremely weak directions and therefore, it is sufficient that it matches the mechanical properties of the surrounding bones as an average [3,4,12,13].

Another important characteristic that must be taken into account for implant design is porosity control. Porosity, pore size and pore interconnectivity are vital for the viability of the implant. Higher porosity facilitates the recruitment and penetration of cells from the surrounding tissue and vascularization and thus, benefits bone ingrowth [12,14]. The influence of pore size is not as consensual among researchers as several references determine different pore sizes as optimal for bone ingrowth [12,15,16,17]. The specific surface area of scaffold is also highly relevant, as high surface areas imply a larger area for bone tissue ingrowth, and scaffolds with smaller pores will present higher surface area [12,18]. However, a higher surface area implies larger frictional forces, which is an obstacle to permeability [3]. The transportation of cells, nutrients, and growth factors require blood flood in the scaffold and thus, permeability is relevant in the design of scaffold [3,12].

In 2007, Harrysson et al. [19] mentioned in their work the possibility of using optimization algorithms to develop non-stochastic porous implants to improve the properties of the femoral stem. Since then, several works followed, with some examples of functionally graded porous cementless femoral stems. In the work of Brian et al. [20], the functional gradient was prescribed axially and radially instead of being calculated from optimization analysis.

Based on the body-centered cubic (BCC) structure, Alkhatib and Tarlochan [21] also developed femoral stem implants with graded density. In their work, the gradient consisted of layers with different densities.

Functionally graded scaffolds for stress shielding minimization may be obtained using optimization algorithms based on bone remodeling. Munteanu et al. [22], Orellana et al. [23] used structural optimization to reduce the stiffness of the femoral stem. However, in their work, the implant is not porous.

In the works of Pais et al. [24,25], which consisted on the development of porous implants, it was shown that the bio-inspired algorithm combined with an experimental material law for the gyroid infill obtained through 3D printing (FFF) [26,27] achieved structural configuration with adequate stiffness to replace human bone.

## 2. Homogenization Techniques

Multiscale modeling refers to an approach in which analysis of the material is conducted at one length scale but the outcomes of the analysis are referent to several properties of the material at another length scale [28].

The use of numerical homogenization techniques allows for significant savings in computational time. Often in composites, it is not necessary (and inefficient) to model the entire structure of the composite. Instead, only a representative region is chosen to model all the constituents of the composite [29]. This approach can be extended to lattice materials, by simplifying the assumption, where the composite presents two or more phases (fiber and matrix), and the lattice will only present one phase, being the rest a void phase. In summary, the porous material is transformed into an equivalent solid material with homogenized properties [30].

The mechanical properties of the obtained scaffold are given as a function of their relative density. The relative density ρ∗ of the scaffold is given by

(3) $$\rho *=\frac{\rho }{\rho_{m}}$$

where ρ is the density of the lattice and $\rho_{m}$ denotes the density of the constituent material. Therefore, from now on, whichever property is being studied, it being stiffness or strength, it is presented as a dependency on this parameter.

When the lattice unit cell presents cubic symmetry, which occurs for most cases indicated in the previous section, the stiffness tensor C will only present three independent constants, $C_{11}$, $C_{12}$ and $C_{44}$. Therefore, the stress-strain relation can be given as:

(4) $$\left[\begin{array}{c} \sigma_{11}\\ \sigma_{22}\\ \sigma_{33}\\ \sigma_{12}\\ \sigma_{23}\\ \sigma_{13} \end{array}\right]=C\left[\begin{array}{c} \varepsilon_{11}\\ \varepsilon_{22}\\ \varepsilon_{33}\\ \varepsilon_{12}\\ \varepsilon_{23}\\ \varepsilon_{13} \end{array}\right]$$

where $\sigma_{ij}$ are the components of the macroscopic stress tensor and $\varepsilon_{ij}$ are the macroscopic strain tensor components.

### 2.1. Boundary Conditions

The most correct way to evaluate the mechanical properties of the lattice material by analyzing one unit cell is through the application of periodic boundary conditions, instead of uniform tensile or linear displacement boundary conditions. The formulations for some possible boundary conditions are presented next.

#### 2.1.1. Periodic Boundary Conditions

**Figure 2:**
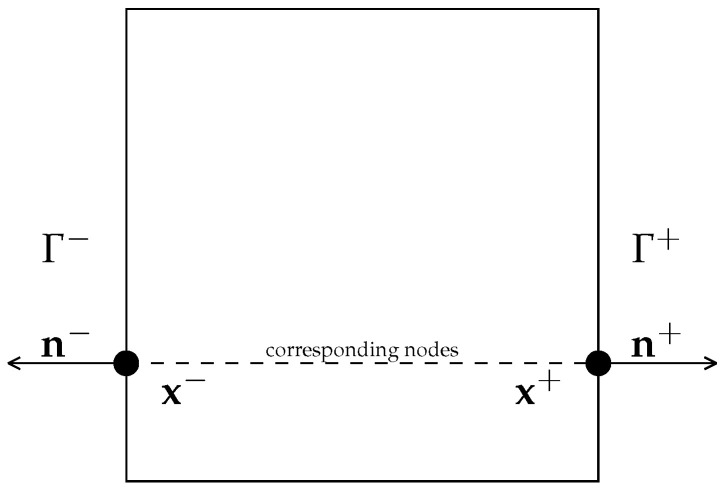
Periodic boundary conditions on the RVE.

Considering a periodic RVE (Figure 2) Ω, the boundary Γ of the RVE can be decomposed into two parts $\Gamma^{+}$ and $\Gamma^{-}$. Each point $x^{+}$ on $\Gamma^{+}$ is connected to just one unique point $x^{-}$ on $\Gamma^{-}$ and the normal vectors to each point are symmetrical so that $n^{+}=-n^{-}$ [29].

The local displacement field u can be decomposed as

(5) $$u\left(x\right)=\hat{u}\left(x\right)+\tilde{u}\left(x\right)=u_{0}+H\cdot x+\tilde{u}\left(x\right)$$

where the macroscopic displacement gradient H is equal to the macroscopic strain ε up to a rotation, $\hat{u}=u_{0}+H\cdot x$ is the macrodisplacement that corresponds to the applied strain, and $\tilde{u}\left(x\right)$ is a micro-displacement. The unit cell simulations assume that in the bulk of the material

(6) $$\tilde{u}\left(x+a\cdot \lambda_{i}\cdot e_{i}\right)=\tilde{u}\left(x\right)$$

for any integer $\lambda_{i}$ and all positions of x, and where *a* denotes the unit-cell length and $e_{i}$ denotes the principal directions. Thus, it is assumed that the micro-displacement field shares the periodicity of the lattice [31].

The periodicity of micro-displacements is enforced by kinematically constraining the difference in the displacements of paired nodes, and setting this difference as equal to the displacement deduced from the macroscopic strain [31] so that

(7) $$u\left(x^{+}\right)-u\left(x^{-}\right)=a\cdot H\cdot n$$

#### 2.1.2. Linear Displacement and Uniform Traction Boundary Condition

Linear displacement boundary conditions consist of applying to the boundary of the RVE the displacement field that would occur if the strain were uniform inside the RVE

(8) u=〈ε(u)〉xonΓ

where ε(u) is the micro strain and x is the position vector on the boundary ∂Ω. This method presents the advantage of having no restriction to its application except that no rigid part must intersect the boundary [32].

Uniform traction boundary conditions consist of applying on the boundary of the RVE the stress vector field that would occur if the stress were uniform inside the RVE

(9) σn=〈σ〉nonΓ

where σ is the Cauchy stress tensor. Using this method, there is also no restrictions except that no holes must intersect the boundary [32].

### 2.2. FE Homogenization

The finite element (FE) homogenization technique can be used in order to predict the effective elastic, as well as the elastic-plastic properties of the material. This technique excels at obtaining the properties of materials with complex microstructures, such as composites [29], even though in this case the focus is on lattice materials.

Considering the RVE Ω, any micro-field f, such as stress or strain within the RVE can have the following average functions defined

(10) $$\langle f\rangle =\frac{1}{V}\int_{\Omega }f\left(x\right)dx$$

where *V* is the volume of the lattice.

The effective mechanical properties do not depend on the body forces or boundary conditions. Thus, to predict the properties of the material, the following weak-form quasi-static equilibrium differential equation is considered

(11) $$\nabla \left(\sigma \left(x\right)\right)=0\;in\;\Omega$$

The boundary conditions do not affect the material properties but they must satisfy Hill’s energy law, which states that the energy on the micro-level has to be the same as the effective energy for the homogenized material

(12) 〈σ:ε〉=〈σ〉:〈ε〉

For any point of the RVE, the constitutive model is given as

(13) σ(x)=σ(x,ε(x))

Based on this last relationship, as well as the quasi-static equilibrium differential equation and the boundary condition which satisfies the Hill energy principle, the stress σ(x) and strain ε(x) can be obtained through FE analysis and then the average values for stress and strain can be computed through

(14) $$\langle \sigma_{ij}\rangle =\frac{1}{V}\sum_{e=1}^{n_{e}}V_{e}\left[\sum_{I=1}^{n_{q}}\sigma_{ij}\left(y_{I}\right)\cdot J\left(y_{I}\right)\cdot W\left(y_{I}\right)\right]$$

(15) $$\langle \varepsilon_{ij}\rangle =\frac{1}{V}\sum_{e=1}^{n_{e}}V_{e}\left[\sum_{I=1}^{n_{q}}\varepsilon_{ij}\left(y_{I}\right)\cdot J\left(y_{I}\right)\cdot W\left(y_{I}\right)\right]$$

where $n_{e}$ is the number of elements in the model, $n_{q}$ is the number of integration points in the element *e* and $W(y_{I})$ is the weight of the integration point. $\sigma_{ij}\left(y_{I}\right)$ and $\varepsilon_{ij}\left(y_{I}\right)$ are the stress and strain respectively, evaluated at the integration point $y_{I}$.

The average values, which consist of a volume average of the properties along the material, can then be used to calculate the effective elastic stiffness tensor C and the effective stress tensor for elastic-plastic analysis.

### 2.3. Mechanical Properties

#### Elastic Properties

The elastic constants are obtained from the following relations from the elastic constants $C_{11}$ and $C_{12}$

(16) $$E^{*}=C_{11}-\frac{2C_{12}^{2}}{C_{11}+C_{12}}$$

(17) $$\nu^{*}=\frac{C_{12}}{C_{11}+C_{12}}$$

(18) $$G^{*}=C_{44}≃\frac{E^{*}}{2(1+\nu^{*})}$$

where each component of the constitutive matrix is obtained from the linear elastic analysis. By enforcing a unit strain, the stress tensor will correspond to one line in the constitutive matrix C.

The isotropy of the surface may be more easily perceived using Young’s modulus surface, which is a representation of Young’s modulus in every direction. For a perfectly isotropic material, where Young’s modulus will be the same in all directions, the surface should have the appearance of a sphere. The isotropy of the structure is proved by the Zener ratio $A^{H}$, which allows the near equality in Equation (18). If $A^{H}$ is close to 1, the structure is isotropic [33].

(19) $$A^{H}=\frac{2C_{44}}{C_{11}-C_{12}}$$

### 2.4. Plastic Properties

Unlike elastic deformation, plastic deformation is irreversible when the loading is removed. When the material reaches its yield point, the material starts displaying plastic behavior and lower material stiffness. After reaching this point, plastic behavior may follow several different models.

If the material is assumed to display linear strain-hardening characterized by the tangential modulus $E_{T}$. The total deformation caused by a stress increase dσ is given by

(20) $$d\varepsilon =d\varepsilon_{p}+d\varepsilon_{e}$$

and so a strain-hardening parameter $H^{\prime }$ is defined as

(21) $$H^{\prime }=\frac{d\sigma }{d\varepsilon_{p}}$$

which can also be given by

(22) $$H^{\prime }=\frac{d\sigma }{d\varepsilon -d\varepsilon_{e}}=\frac{E_{T}}{1-E_{T}/E}$$

which corresponds to the slope of the strain-hardening portion of the stress-strain curve [34].

In the elastic domain, the stiffness exhibited by the material will be given by

(23) $$K_{e}=\frac{F}{\delta }=\frac{E\cdot A}{L}$$

where *F* is the applied force, δ is the shown displacement, *E* is the Young’s modulus, *A* is the cross-sectional area and *L* is the length. Thus, the stiffness matrix for an element is

(24) $$K_{e}^{\left(e\right)}=\frac{E\cdot A}{L}\left[\begin{array}{cc} 1 & -1\\ -1 & 1 \end{array}\right]$$

If the material reaches its yield stress due to an increase in *F*, that increase dF will generate a dδ of

(25) $$d\delta =\left(d\varepsilon_{e}+d\varepsilon_{p}\right)L$$

Considering that the increase in force is given by

(26) $$dF=A\cdot d\sigma =A\cdot H^{\prime }\cdot d\varepsilon_{p}$$

the tangential stiffness of the material will be

(27) $$K_{ep}=\frac{dF}{d\delta }=\frac{A\cdot H^{\prime }\cdot d\varepsilon_{p}}{L\left(d\sigma /E+d\varepsilon_{p}\right)}=\frac{E\cdot A}{L}\left(\;-\frac{E}{E+H^{\prime }}\right)$$

which leads to an element stiffness of

(28) $$K_{ep}^{\left(e\right)}=\frac{E\cdot A}{L}\left(1-\frac{E}{E+H^{\prime }}\right)\left[\begin{array}{cc} 1 & -1\\ -1 & 1 \end{array}\right]$$

Finally, the equivalent yield strength of the lattice is given by the apparent stress-strain curve, by retrieving the stress level that leads to 0.2% plastic strain. The apparent stress (macro-stress) $\sigma_{11}$ is obtained as

(29) $$\sigma_{11}=\frac{\sum F_{x}^{l=l_{RVE}}}{A_{RVE}}$$

while the apparent strain (macro-strain) is given by:

(30) $$\varepsilon_{11}=\frac{u_{1}^{l=l_{RVE}}}{l_{RVE}}$$

### 2.5. Scaling Laws

Cellular materials are usually described by scaling laws which correlate the homogenized mechanical properties to the volume fraction of the cell [35]. The volume fraction of the cell is given by

(31) $$\frac{\rho^{*}}{\rho_{s}}=\frac{V*}{V_{RVE}}$$

The homogenized Young’s modulus is given by

(32) $$\frac{E^{*}}{E_{s}}=D_{1}{\left(\frac{\rho^{*}}{\rho_{s}}\right)}^{n}$$

and the homogenized yield stress is given by

(33) $$\frac{\sigma^{*}}{\sigma_{s}}=D_{2}{\left(\frac{\rho^{*}}{\rho_{s}}\right)}^{n}$$

The *n* coefficient depends on whether the cell exhibits stretching-dominated or bending-dominated behavior. The stretching/bending dominated designation is referent to the deformation mode of cell walls. Usually, higher density foams will tend to show near stretching-dominated behavior while at lower densities these will tend to show bending-dominated behavior. Table 1, adapted from the review paper by [36] summarizes the *n* coefficient in Equations (32) and (33), while the $D_{1}$ coefficient will range between $\left[0.1,4\right]$ and the $D_{2}$ coefficient will range between $\left[0.1,1\right]$ [36].

In the literature, however, it is common to find modifications to the Gibson-Ashby model, where none of the values shown in Table 1 are necessary. It’s also relevant to mention that this model will change depending on the manufacturing process, material, and geometry of the cells. For some complex materials characterized as a function of their relative density, the fitting to the model allows determining if the structure exhibits bending or stretching-dominated behavior.

In this work, the correlations will describe the homogenized mechanical properties using polynomial models as shown in (34) and (35)

(34) $$\frac{E^{*}}{E_{s}}=a_{1}{\left(\frac{\rho^{*}}{\rho_{s}}\right)}^{3}+a_{2}{\left(\frac{\rho^{*}}{\rho_{s}}\right)}^{2}+a_{3}\left(\frac{\rho^{*}}{\rho_{s}}\right)+a_{4}$$

(35) $$\frac{\sigma^{*}}{\sigma_{s}}=b_{1}{\left(\frac{\rho^{*}}{\rho_{s}}\right)}^{3}+b_{2}{\left(\frac{\rho^{*}}{\rho_{s}}\right)}^{2}+b_{3}\left(\frac{\rho^{*}}{\rho_{s}}\right)+b_{4}$$

## 3. Materials and Methods

### 3.1. Materials

For the homogenization analysis, each unit cell model presents a Young’s modulus equal to 3000 MPa and a Poisson ratio equal to 0.3. For the plasticity analysis, the material is considered to be elastic-perfectly-plastic, being that three different yield stress values were tested, 10 MPa, 30 MPa and 50 MPa.

### 3.2. Models

The STL models of each surface were created in MATLAB by computing the equation with an isolevel value *t* equal to zero. Each facet in the triangular mesh was projected in its normal direction by half of the thickness value, in the positive and negative directions. The solid model was meshed into quadratic 10-node tetrahedral elements from the STL of the outer surface. For both the gyroid and the primitive three models were obtained, each corresponding to a low density, medium density, and high density. Each density level corresponds to a different wall thickness to RVE edge length ratio, as can be visualized in Table 2.

### 3.3. Boundary Conditions

For the elastic analysis, two different types of boundary conditions were used, namely periodic boundary conditions and homogenization boundary conditions.

#### 3.3.1. Periodic Boundary Conditions

The enforcement of periodic boundary conditions required the definition of paired nodes in the faces. Each node on one face is paired with the closest node on the opposite face. Due to the fact that both faces do not present the same number of nodes, some nodes do not have any equation constraint associated with them. The primitive structure unit cell does not have edges or vertices, while the gyroid structure has edges and vertices. The nodes on the edges and vertices in the gyroid model were ignored for the enforcement of periodic boundary conditions.

#### 3.3.2. Homogenization Boundary Conditions

These boundary conditions, adapted from [37] were used both in the elastic and plastic analysis. If a normal strain is applied, for example $\varepsilon_{11}$, the following displacements are imposed:

$$u_{1}^{x^{+}}=\varepsilon_{11}\wedge u_{1}^{x^{-}}=0\wedge u_{2}^{y^{+}}=0\wedge u_{2}^{y^{-}}=0\wedge u_{3}^{z^{+}}=0\wedge u_{3}^{z^{-}}=0$$

whereas if a shear strain is applied, for example $\varepsilon_{12}$, the following displacements are imposed

$$u_{2}^{x^{+}}=\varepsilon_{12}/2\wedge u_{2}^{x^{-}}=0\wedge u_{1}^{y^{+}}=\varepsilon_{12}/2\wedge u_{1}^{y^{-}}=0\wedge u_{3}^{z^{+}}=0\wedge u_{3}^{z^{-}}=0$$

The application of these boundary conditions ensures that the prescribed displacement in the top of the RVE is a unit displacement and all other components of the strain tensor remain zero.

### 3.4. Bio-Inspired Remodelling Algorithm

In biomedical implants, stress shielding can be minimized as the stiffness of the implant is optimized according to the bone stiffness [13].

**Figure 3:**
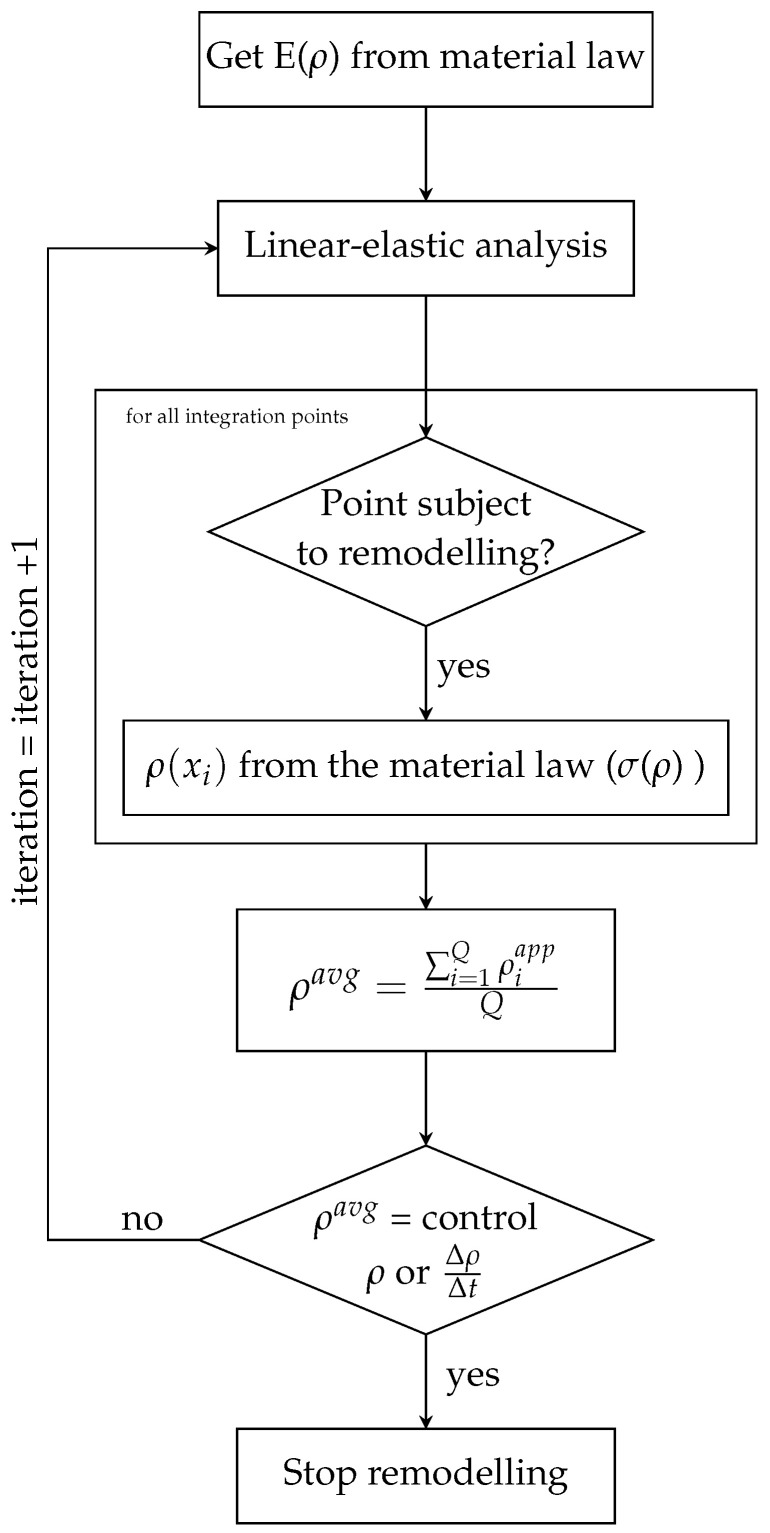
Flowchart for the bio-inspired remodelling algorithm.

The bio-inspired remodelling, taking into consideration that bone remodelling phenomena acts as structural optimization [38], updates the density of the structure iteratively. Therefore, the bio-inspired algorithm, based on [39] can be summarized according to Figure 3.

In summary, first, the mechanical properties of a point are determined from $E\left[MPa\right]=f_{E}\left(\rho \left[g/cm^{3}\right]\right)$ so the constitutive matrix is obtained and then, linear-elastic analysis is performed. Using scaling laws, the correlations are adjusted to the material of the implant. Then, the critical variable is used in order to determine which points must be remodelled, meaning, having its density increased or decreased. Points with higher stress or strain energy density can have its density increased and points with lower stress or strain energy density may have its density decreased. As verified in bone remodelling phenomenon (in which unsolicited bone will decay while a stress stimulus promotes bone growth), in tihs bio-inspired algorithm points with the highest values of the critical variable will increase its density and the points with the lowest values of the critical variable will have its density decreased. This density update is enforced using $\sigma \left[MPa\right]=f_{\sigma }\left(\rho \left[g/cm^{3}\right]\right)$. The remodelling process occurs until convergence, or, until the control density defined by the user is achieved. Figure 3 shows a schematic flowchart of the remodelling algorithm.

### 3.5. Stress Shielding Evaluation

Stress shielding is usually evaluated as a function of the mismatch between the stresses in the bone and stresses in the implant [40].

(36) $$SS\left[\%\right]=\frac{\sigma_{bone}-\sigma_{implant}}{\sigma_{bone}}\times 100\%$$

In order to avoid stress shielding, the stiffness of the implant must be lower than the stiffness of bone. Therefore, the loads transferred to the bone are higher, resulting in a stress stimulus leading to bone growth. In order to establish a term of comparison, a model with the geometry of the area being remodelled in the implant is modeled as bone, through a bone remodelling algorithm [39]. The stiffness of that bone, is then compared to the stiffness of the implant.

The stiffness of the implant is evaluated as follows:

(37) $$K_{z}\left[N/mm\right]=\frac{f[N]}{d[mm]}$$

where

(38) $$f=\sum_{i=1}^{n_{t}}F_{z}^{t}$$

and

(39) $$d=\frac{\sum d_{z}^{t}}{n_{t}}$$

where *t* refers to the nodes where the load is applied and $n_{t}$ is the number of nodes where the load is applied. Because at each iteration the variables are scaled to the elastic limit of the porous material, the applied force is calculated from the stress field as:

(40) $$f=\int B^{T}\sigma d\Omega$$

where B is the deformability matrix and σ is the stress tensor in Voigt notation.

## 4. Results and Discussion

### 4.1. Topology Analysis

**Figure 4:**
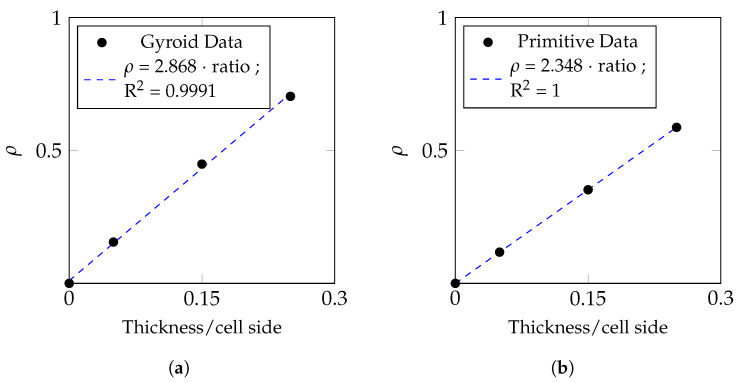
Relative density of the gyroid (**a**) and primitive (**b**) structures as a function of its geometric parameters: unit cell size and wall thickness.

Figure 4 shows the plot of the relative density of the cell as a function of its thickness and unit cell length. For the same wall thickness, the gyroid presents a higher relative density than the primitive structure.

### 4.2. Elastic Properties

The elastic properties of both structures can be consulted in Table 3. The gyroid structure presents higher Young’s modulus and higher isotropy than the primitive structure. Additionally, the use of periodic boundary conditions in the gyroid structure led to higher Young’s modulus values than the primitive structure.

### 4.3. Plastic Properties

**Figure 5:**
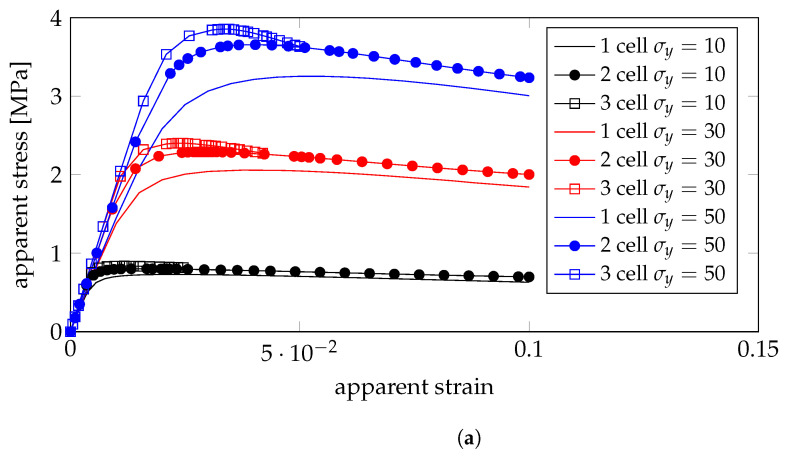

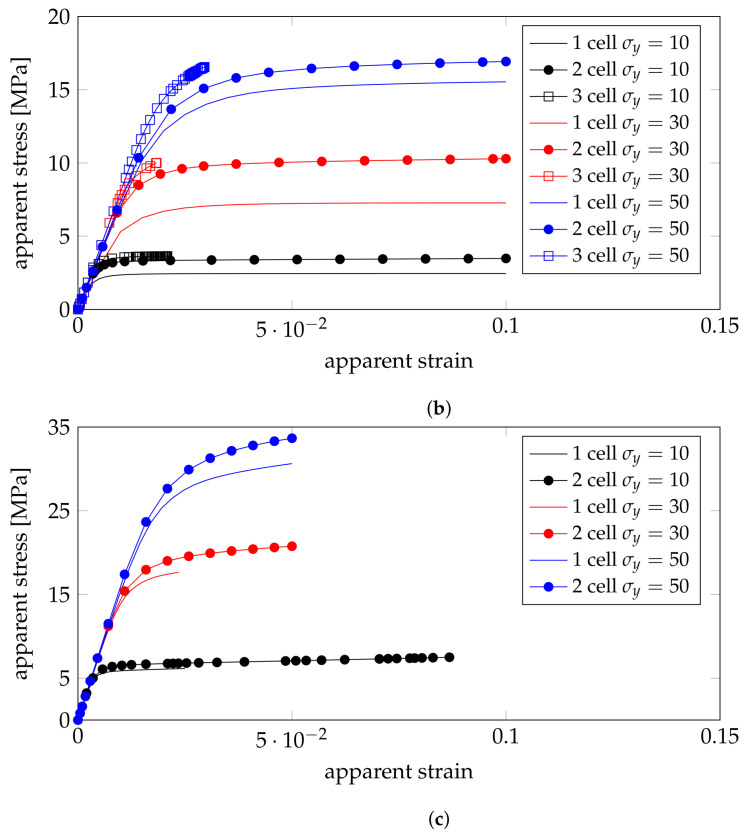
Apparent stress-strain plots using linear displacement boundary conditions on the gyroid model: (**a**) low density; (**b**) medium density; (**c**) high density.

**Figure 6:**
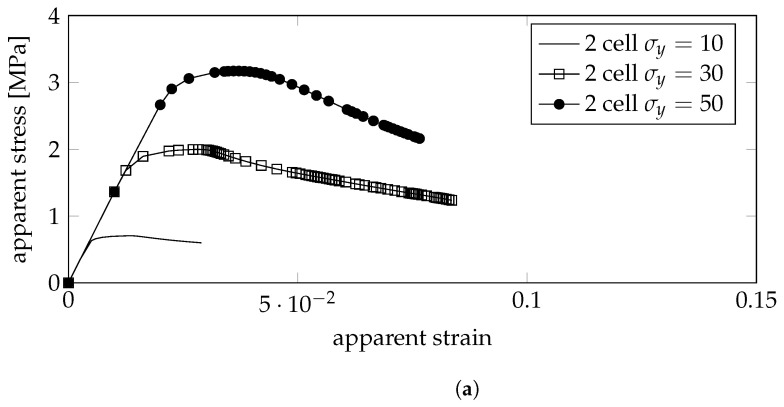

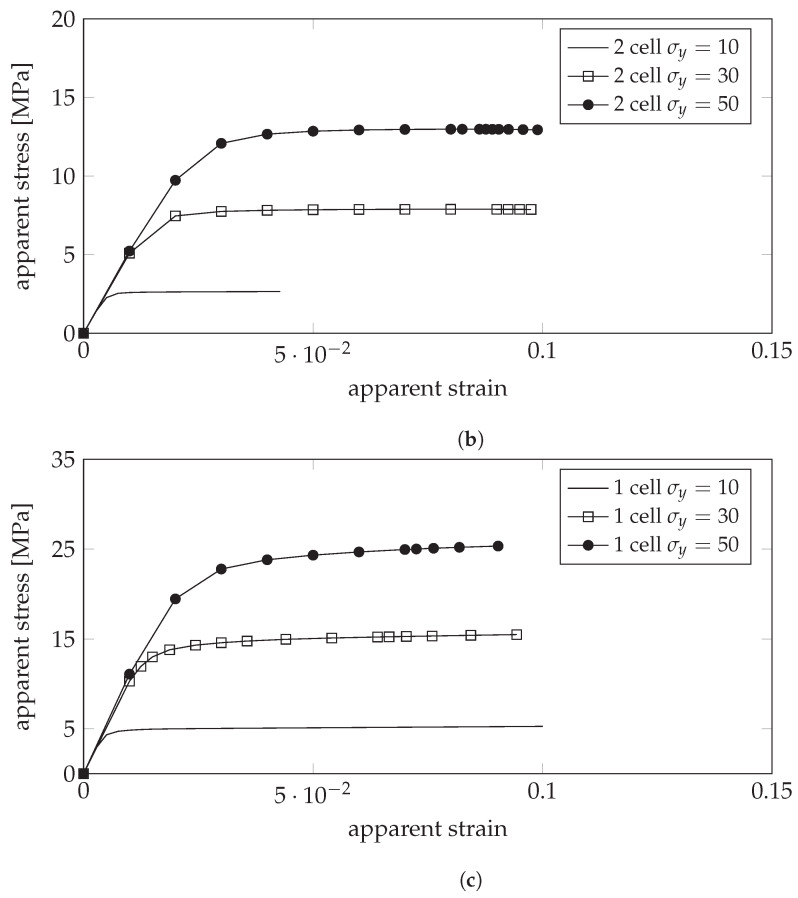
Apparent stress-strain plots using linear displacement boundary conditions on the primitive model: (**a**) low density; (**b**) medium density; (**c**) high density.

For the gyroid, the apparent stress-strain plots are shown in Figure 5 for RVEs consisting of 1, 2, and 3 unit cells on each side, while for the primitive structure, only 2 by 2 cells were tested, where the results are shown in Figure 6. It can be concluded that the gyroid structure presents higher strength than the primitive structure. For both geometries, it can be seen that higher-density foams exhibit hardening (near positive slope in the plastic domain of the stress-strain curve) while medium-density foams present near perfectly plastic behavior (near zero slopes in the plastic domain of the stress-strain curve) and lower density foams present a yield point.

Because the aim of the plastic analysis is to obtain the elastic limit of the material, these analyses only consisted of a maximum strain of 10%.

In order to develop material laws that can be used in the bio-inspired algorithm, the apparent yield stress was obtained from the apparent stress-strain curves, resulting in the data shown in Table 4, Table 5 and Table 6 for the gyroid structure and Table 7 for the primitive structure.

## 5. Discussion

### 5.1. Material Law and Comparison to Literature Data

By adjusting the obtained points to a third degree polynomial function, the coefficients in Equations (34) and (35) are obtained, which can be consulted in Table 8.

Both structures exhibit bending-dominated behavior instead of stretching-dominated behavior, meaning that the cell walls tend to bend instead of being compressed. Stretching-dominated structures tend to be stiffer which can be an issue regarding the minimization of stress shielding. This is in agreement with other studies found in the literature, such as the work of Abueidda et al. [41] where gyroid sheet foams were modeled to fit the Gibson-Ashby model and it was concluded from the coefficients of that model that the structure exhibits bending-dominated behavior. Al-Ketan et al. [5] also concluded that these sheet-like structures exhibit behavior between stretching and bending dominated.

The developed material law assumes that the material is isotropic and perfectly plastic. From the analysis of elastic properties in Table 3 it was concluded that the primitive structure is less isotropic than the gyroid structure.

**Figure 7:**
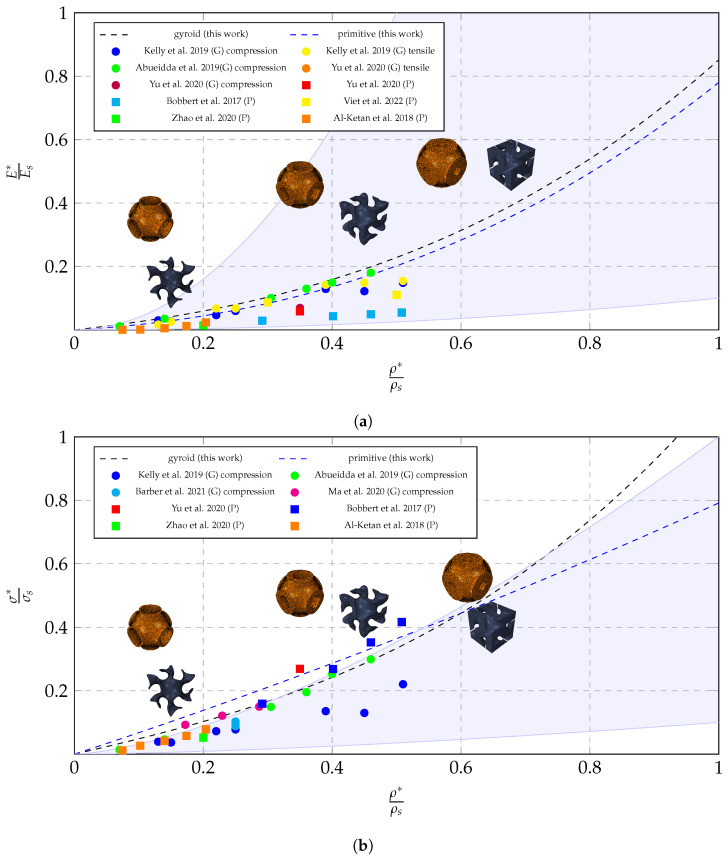
Comparison of the obtained scaling law with other works from the literature ([3,41,42,43,44,45,46,47,48]) and its localization regarding the bounds defined by the Gibson-Ashby model: (**a**) relative elastic modulus and and (**b**) relative yield stress.

Figure 7 shows the developed laws in comparison to the several works from experimental testing in the literature. Most experimental works use models with a higher number of unit cells than this work. It can be seen from the figures that the error in homogenized modulus is, for most cases, low, and thus, the material law will accurately describe the mechanical properties of gyroid foams at several densities. Moreover, this highlights the importance of experimental testing in the validation and calibration of the material law.

The geometry presented by these complex materials is almost always unattainable through conventional manufacturing, leaving additive manufacturing as the only solution to obtain physical parts or prototypes. However, additively manufactured parts tend to be highly anisotropic, showing different mechanical properties in the building direction. The developed correlations do not account for the anisotropy in the manufacturing process. The anisotropy can be minimized by choosing a process with higher isotropy, for example, SLM or SLS if dealing with metals or polymers respectively, instead of extrusion-based processes, where the interlayer bonding effect highly influences the mechanical properties of printed parts.

Additionally, the plot shows, through the shaded area the upper and lower bounds of the Gibson-Ashby law, according to the work of Maconachie et al. [36]. The Gibson-Ashby model, which predicts the coefficients for metallic foams, was developed based on analytical models, extensive testing on polymeric foams, and empirical fits to experimental data [36,49]. Finally, in order to evaluate the accuracy of the developed models in comparison to the literature, the mean absolute percentage error was calculated for each of the models (Young’s modulus and yield stress for both unit cells). These values are presented in Table 9.

### 5.2. Study Case-Femoral Stem

In order to establish conclusions regarding the suitability of the gyroid material implant, remodeling analysis was performed on a cementless femoral stem model. These implants are prone to stress shielding, and therefore, the use of a porous structure can reduce stiffness and enhances bone ingrowth [40]. Previously, it was established that the gyroid foam is more isotropic than the primitive foam. Thus, as the remodeling model assumes an isotropic material, the gyroid material model is used.

#### Implant Model

The femoral stem was modeled according to geometries found in the literature using CAD software and then meshed into linear hexahedral elements. The final mesh has 2808 nodes and 2020 elements.

**Figure 8:**
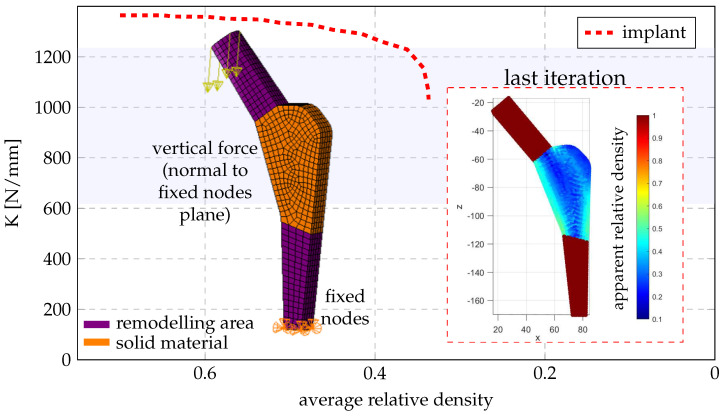
Implant model indicating the areas that are subject to remodeling and density field at the last iteration. The blue shaded area indicates the range of stiffness values that trabecular bone presents, between apparent densities of 0.4186 and 0.4225.

Only the proximal area of the stem implant is remodeled, the neck and taper area as well as the distal part of the stem remains as solid material (Figure 8). The chosen base material is Ti-6Al-4V alloy whose properties are shown in Table 10, as well as a comparison to femoral trabecular bone, which is used later as reference. The base of the implant was fixed while a vertical force was applied to the top of the taper. This force was set as unitary as at each iteration of the remodeling algorithm the variables are brought to the elastic limit.

### 5.3. Optimized Implant

Figure 8 shows the evolution in stiffness of the implant and average relative density along the iterations of the remodeling algorithm. The final density distribution is also presented at the central cross-section.

**Figure 9:**
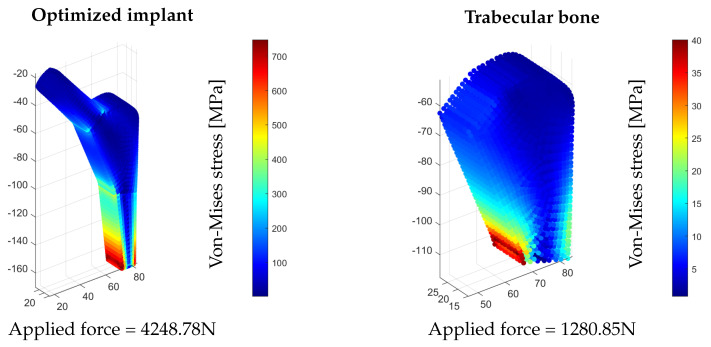
Von-Mises stress fields in the implant and bone, indicating enough load-bearing capacity in the implant.

Finally, in order to evaluate the suitability of the implant, its strength must also be enough to support the loads in the bone. Figure 9 shows that the load that leads the trabecular to its elastic limit is lower than the load that leads the implant to its elastic limit. The force that takes the implant to the elastic limit is 4248.78N, while the force that can be applied to the bone while remaining below the elastic limit is 1280.85N. Thus, the implant is also suitable in terms of strength.

## 6. Conclusions

With this work it was possible to correlate the mechanical properties of two different TPMS, the gyroid and the primitive with the apparent density. The simulations were run considering isotropic and perfectly plastic properties of the solid material and it was verified that this assumption works as a reasonable approximation in comparison to literature data from experimental tests. Additionally, it was shown that the remodelling algorithm provided an optimal configuration for a cementless femoral stem implant based on the gyroid structure. Future directions for the study should include the experimental testing of implant prototypes for final validation of the material law and remodelling algorithm.

## Figures and Tables

**Table 1:** Exponent coefficient *n* in the Gibson-Ashby model for stretching-dominated (SD) and bending-dominated (BD) structures.

	SD	BD
Homogenized Young’s modulus	1	2
Homogenized yield stress	1	1.5

**Table 2:** Wall thickness to unit cell length ratio for each model type.

Model	t/L
low density	0.05
medium density	0.15
high density	0.25

**Table 3:** Summary of the elastic constants and properties of the structure (H—homogenization boundary conditions, P—periodic boundary conditions).

			E*	ν *	**C11**	**C12**	C44 = G*	$A^{H}$
primitive 1 cell	low dens	H	61.83179	0.405701	138.6081	94.62162	48.49176	2.204851
		P	61.75457	0.405275	137.9524	94.0076	63.79892	2.903592
	med dens	H	327.7807	0.350119	526.3416	283.5624	198.3359	1.633879
		P	326.2036	0.349605	522.6321	280.929	222.1894	1.838532
	high dens	H	818.1541	0.30575	1119.694	493.1158	396.8583	1.266748
		P	812.4441	0.305092	1109.737	487.2183	423.5954	1.360908
			E*	ν *	**C11**	**C12**	C44 = G*	$A^{H}$
gyroid 1 cell	low dens	H	113.7596	0.300448	153.3305	65.85327	39.45371	0.902034
		P	155.2857	0.326205	226.9771	109.8868	65.58988	1.12033
	med dens	H	560.7722	0.278805	714.874	276.3613	232.5266	1.060524
		P	608.784	0.295178	808.7353	338.6964	275.3023	1.171402
	high dens	H	1243.97	0.270049	1554.597	575.1302	519.8329	1.061461
		P	1314.799	0.277671	1671.664	642.6045	572.3806	1.112434
			E*	ν *	**C11**	**C12**	C44 = G*	$A^{H}$
gyroid 2 cells	low dens	H	127.534	0.308422	175.9314	78.45974	47.83487	0.98151
	med dens	H	578.4656	0.283865	746.4448	295.8789	252.1427	1.11923
	high dens	H	1292.918	0.270515	1617.423	599.7905	555.19810	1.09116
			E*	ν *	**C11**	**C12**	C44 = G*	$A^{H}$
gyroid 3 cells	low dens	H	142.8026	0.312171	199.2662	90.43695	57.86529	1.063414
	med. dens	H	622.4885	0.285833	807.1683	323.0553	274.9201	1.135768

**Table 4:** Summary of plastic properties of the gyroid structure—low density.

	Low Density								
	1 × 1 × 1			2 × 2 × 2			3 × 3 × 3		
	$\sigma_{y}=10$	$\sigma_{y}=30$	$\sigma_{y}=50$	$\sigma_{y}=10$	$\sigma_{y}=30$	$\sigma_{y}=50$	$\sigma_{y}=10$	$\sigma_{y}=30$	$\sigma_{y}=50$
apparent $\sigma_{y}$	0.729344	2.058199	3.253642	0.801418	2.291044	3.656217	0.836503	2.401517	3.855031
apparent $\sigma_{y}$ ratio	0.072934	0.068607	0.065073	0.080142	0.076368	0.073124	0.08365	0.080051	0.077101
mean ratio	0.068871			0.076545			0.080267		

**Table 5:** Summary of plastic properties of the gyroid structure—medium density.

	Medium Density								
	1 × 1 × 1			2 × 2 × 2			3 × 3 × 3		
	$\sigma_{y}=10$	$\sigma_{y}=30$	$\sigma_{y}=50$	$\sigma_{y}=10$	$\sigma_{y}=30$	$\sigma_{y}=50$	$\sigma_{y}=10$	$\sigma_{y}=30$	$\sigma_{y}=50$
apparent $\sigma_{y}$	2.213404	7.271915	12.18746	3.059191	8.480854	13.66536	3.481273	9.110942	16.54715
apparent $\sigma_{y}$ ratio	0.22134	0.242397	0.243749	0.305919	0.282695	0.273307	0.348127	0.303698	0.330943
mean ratio	0.235829			0.287307			0.327589		

**Table 6:** Summary of plastic properties of the gyroid structure—high density.

	High Density								
	1 × 1 × 1			2 × 2 × 2			3 × 3 × 3		
	$\sigma_{y}=10$	$\sigma_{y}=30$	$\sigma_{y}=50$	$\sigma_{y}=10$	$\sigma_{y}=30$	$\sigma_{y}=50$	$\sigma_{y}=10$	$\sigma_{y}=30$	$\sigma_{y}=50$
apparent $\sigma_{y}$	5.6391	15.43433	25.32575	6.380779	17.95709	27.64402	-	-	-
apparent $\sigma_{y}$ ratio	0.56391	0.514478	0.506515	0.638078	0.59857	0.55288	-	-	-
mean ratio	0.528301			0.596509					

**Table 7:** Summary of plastic properties of the primitive structure.

	Low Density			Medium Density			High Density		
	2 × 2 × 2			2 × 2 × 2			1 × 1 × 1		
	$\sigma_{y}=10$	$\sigma_{y}=30$	$\sigma_{y}=50$	$\sigma_{y}=10$	$\sigma_{y}=30$	$\sigma_{y}=50$	$\sigma_{y}=10$	$\sigma_{y}=30$	$\sigma_{y}=50$
apparent $\sigma_{y}$	0.7032	1.9971	3.1698	2.5376	7.887468	12.98112	4.718293	13.00106	19.45738
apparent $\sigma_{y}$ ratio	0.07032	0.06657	0.063396	0.25376	0.262916	0.259622	0.471829	0.433369	0.389148
mean ratio	0.066762			0.258766			0.431449		

**Table 8:** Coefficients of the polynomial scaling laws relative to the Young’s modulus (a${}_{i}$) and yield stress (b${}_{i}$) for the gyroid and primitive structures.

	Gyroid	Primitive		Gyroid	Primitive
a${}_{1}$	0.1018	0.1766	b${}_{1}$	0.5545	0.0000
a${}_{2}$	0.4388	0.4879	b${}_{2}$	0.1250	0.1259
a${}_{3}$	0.2405	0.1157	b${}_{3}$	0.4666	0.6655
a${}_{4}$	0.0000	0.0000	b${}_{4}$	0.0000	0.0000

**Table 9:** Mean absolute percentage error for all developed models.

	Relative Young’s Modulus	Relative Yield Stress
Gyroid	23.973%	26.4787%
Primitive	63.874%	37.1244%

**Table 10:** Mechanical properties of femoral trabecular bone (as reference) and of the metallic alloy used to manufacture bone scaffold.

	Young’s Modulus [MPa]	Yield Stress [MPa]	Poisson Ratio
Femoral trabecular bone	5850	40	0.3
Ti-6Al-4V	110,000	850	0.3

## Data Availability

Not applicable.
